# Surgical decision-making in pediatric intestinal ascariasis: experience from an endemic region

**DOI:** 10.1186/s12893-026-03488-w

**Published:** 2026-06-09

**Authors:** Fayaz Ahmad Najar, Ab Wahid Raina, Sumiya Jaan, Gowhar Nazir Mufti, Aejaz Ahsan Baba, Nisar Ahmad Bhat

**Affiliations:** Sher-i-Kashmir Institute of Medical Sciences, Soura, Srinagar India

**Keywords:** Intestinal ascariasis, Surgical intervention, Enterotomy, Bowel resection

## Abstract

**Background:**

Intestinal ascariasis is the leading cause of pediatric intestinal obstruction in endemic regions. This study aimed to analyze the clinical presentation, diagnostic accuracy, and outcomes of conservative and surgical management of ascariasis-induced intestinal obstruction in children.

**Methods:**

Children aged 2–16 years who presented with intestinal obstruction between May 2022 and May 2025 were prospectively evaluated. Patients with intestinal ascariasis confirmed as the cause of obstruction were included, whereas those with concomitant adhesions, intussusception, or other etiologies of obstruction were excluded. Data on demographics, management strategies, operative details, complications, and hospital stay were analyzed.

**Results:**

Of the 190 children with intestinal obstruction, 120 (63.2%; 95% CI: 56.3–70.0%) were diagnosed with ascariasis-induced obstruction. There was a male predominance (male-to-female ratio 2:1). The mean age at presentation was 5.8 ± 2.3 years, with the highest incidence observed in the 5–8-year age group (54.2%), followed by the 2–4-year age group (37.5%). Initial conservative management was attempted in 80 of the 120 patients, of whom 65 (81.3%; 95% CI: 72.7–89.8%) improved without surgical intervention. Surgical management was required in 55 patients (45.8%; 95% CI: 36.9–54.7%). The procedures performed included manual kneading of worms (36.4%), enterotomy with worm extraction (32.7%), and bowel resection with end-to-end anastomosis (30.9%). Postoperative complications were observed in eight patients (14.5%; 95% CI: 5.4–23.7%), including surgical site infection (7.3%), anastomotic leak (3.6%), and burst abdomen (3.6%). No mortality was observed. The mean length of hospital stay was 4.2 ± 1.5 days for patients managed conservatively and 7.8 ± 1.5 days for those who underwent surgical intervention. Ultrasonography demonstrated a diagnostic accuracy of 96.5% (95% CI: 93.1–99.8%) and PPV of 100% (95% CI: 95.6–100%) among patients who underwent imaging, with characteristic “bull’s-eye” and “railway-track” appearances of intraluminal worms.

**Conclusion:**

Ascariasis remains the predominant cause of pediatric intestinal obstruction in endemic areas, underscoring its persistent public health relevance. Although conservative management is effective in most cases, timely surgical intervention remains essential for patients with complications or failed conservative treatment.

## Introduction

Ascariasis, caused by *Ascaris lumbricoides*, is the most prevalent helminthic infection worldwide, affecting an estimated 1.5 billion individuals, predominantly in low- and middle-income countries [1]. Poor sanitation, inadequate hygiene, and low socioeconomic conditions facilitate sustained transmission, making ascariasis a persistent public health problem in endemic regions [2, 3].

In children, intestinal ascariasis is a leading cause of small bowel obstruction in developing countries, including India, in contrast to developed nations, where postoperative adhesions predominate [4, 5]. The burden is particularly high in the Indian subcontinent, with infestation rates reported in up to 70% of children, rising further in hyperendemic areas such as Kashmir [6]. Pediatric patients are especially vulnerable due to their smaller intestinal lumen, higher relative worm load, and increased risk of complications [7–9].

The clinical spectrum ranges from asymptomatic infestation to acute intestinal obstruction, which represents the most serious surgical complication of ascariasis and is associated with significant morbidity and mortality if treatment is delayed [10]. Obstruction typically results from bolus formation of worms, bowel spasm around the worm mass, inflammatory adhesions, or secondary complications such as volvulus and intussusception, most commonly involving the terminal ileum [11–13].

While the majority of cases respond to conservative management, surgical intervention is required in patients with complete obstruction, bowel ischemia, perforation or peritonitis [14, 15]. Delayed presentation increases the risk of serious complications, including bowel necrosis, perforation, and septic sequelae [16].

Ultrasonography plays a pivotal role in diagnosis, demonstrating characteristic features such as the “bull’s-eye” and “railway track” signs, whereas radiography and computed tomography assist in assessing obstruction severity and associated complications [17, 18]. Surgical management is guided by intraoperative findings and includes kneading of worms into the colon, enterotomy with worm extraction, or bowel resection with primary anastomosis in a compromised bowel [19, 20]. Postoperative complications, although infrequent, may include surgical site infection (SSI) and anastomotic failure [21].

Despite the availability of improved diagnostic and therapeutic modalities, intestinal ascariasis continues to pose significant challenges in endemic regions. A clear understanding of its clinical presentation, management strategies, and surgical outcomes is essential to reduce disease-related morbidity and improve pediatric surgical care.

## Materials and methods

### Study design and setting

This prospective observational study was conducted at a Tertiary Care Center of North India (SKIMS, Soura, Srinagar) over 3 years (2022 to 2025).

This study was designed to evaluate the clinical presentation, management approaches, and surgical outcomes of pediatric patients with intestinal obstruction due to ascariasis over the defined study period.

The study was conducted in accordance with the principles of the Declaration of Helsinki (latest version) and the Good Clinical Practice guidelines. Data collection and manuscript preparation were in compliance with the guidelines set forth by the Committee on Publication Ethics (COPE) and the Strengthening the Reporting of Observational Studies in Epidemiology (STROBE) initiative for reporting cohort studies. Patient confidentiality was maintained throughout the study period, and all data were anonymized for analysis and publication.

### Study population and inclusion criteria

The study population comprised all pediatric patients age group–2–16 years who presented to the emergency department with a clinical diagnosis of intestinal obstruction. Patients were systematically screened upon admission, and those diagnosed with intestinal obstruction secondary to ascariasis were included in the detailed analysis. This age range was specifically chosen as it represents the most vulnerable population for Ascaris-induced complications due to the smaller intestinal diameter and higher susceptibility to worm burden-related obstruction.

### Patient assessment and data collection

All patients underwent comprehensive clinical evaluation. Detailed history taking focused on the presenting symptoms, duration of illness, prior similar episodes, and relevant epidemiological factors. Physical examination included assessment of hydration status and vital signs, followed by a thorough abdominal examination comprising inspection, palpation, percussion, and auscultation of the abdomen. Digital rectal examination was performed in all patients to evaluate palpable masses or evidence of lower gastrointestinal pathology.

### Diagnostic investigations

Baseline laboratory investigations were performed for all patients and included a complete hemogram to evaluate anemia, leukocytosis, and other hematological abnormalities associated with parasitic infestation. Serum electrolytes were assessed to identify dehydration and electrolyte imbalances resulting from vomiting and reduced oral intake. Stool examination for ova or adult worms was performed when feasible but was often limited in patients presenting with complete intestinal obstruction.

Radiological evaluation is a key component of the diagnostic workup. Plain chest radiography was performed to exclude any associated pulmonary pathologies. Abdominal radiographs in the erect and supine positions were obtained to assess features of intestinal obstruction, including dilated bowel loops, air–fluid levels, and, in some cases, visible worm shadows.

Ultrasonography was performed in selected patients using a high-frequency linear transducer (7.5–12 MHz) to identify intraluminal Ascaris boluses, demonstrating characteristic “railway track” and “target” signs. A low-frequency curvilinear probe (3.5–5 MHz) was employed when necessary to evaluate proximal bowel dilatation, level of obstruction, bowel viability, and associated intra-abdominal complications to facilitate surgical decision-making.

In patients with suspected bowel perforation or clinical features of peritonitis, erect chest radiography and/or contrast-enhanced computed tomography (CT) of the abdomen was performed to detect pneumoperitoneum, bowel ischemia, gangrene, and perforation. CT imaging also facilitates the assessment of the extent of bowel involvement and the identification of associated complications, including bowel wall thickening and intra-abdominal fluid collections.

### Management protocol

#### Conservative management

Patients presenting with clinical features suggestive of partial intestinal obstruction, absence of peritoneal signs, and no evidence of free intraperitoneal air on imaging were initially managed conservatively for 24 h. The conservative management protocol included aggressive fluid and electrolyte replacement therapy to correct dehydration and metabolic imbalances, nasogastric decompression to relieve gastric distension and prevent aspiration, broad-spectrum antibiotic coverage to prevent secondary bacterial infections, and appropriate analgesics for pain management of the patients.

Importantly, anti-helminthic drugs were not administered during the acute phase of obstruction in this study, as these medications can potentially worsen obstruction by increasing the size and activity of the worm bolus, leading to more severe impaction. Some centers have reported the use of hypertonic saline enemas as an adjunctive measure, although this was not routinely employed in our protocol.

The success of conservative management was defined clinically when a patient had stabilization of vital signs, such as pulse, respiratory rate, and temperature; resolution of vomiting and abdominal pain; reduction of abdominal distension and tenderness; evidence of passage of stools and worms; and successful oral tolerance.

Failure of conservative management was defined as the development of clinical or radiological signs suggestive of bowel compromise, including tachycardia, tachypnea, fever, increasing abdominal tenderness, persistent or progressive abdominal distension, ongoing vomiting, features of peritonitis, and the presence of fixed bowel loops on serial clinical examinations or follow-up abdominal radiographs, and/or radiological evidence of free intraperitoneal air.

### Surgical management

Surgical intervention was performed in patients who failed to respond to conservative management after 24–48 h, those presenting with complete intestinal obstruction, clinical features of peritonitis, or radiological evidence of bowel perforation or pneumoperitoneum. The decision to proceed with surgery was guided by clinical deterioration, including persistent fever, worsening abdominal distension, rising leukocyte counts, or overall clinical decline despite adequate conservative therapy.

The choice of surgical procedure was determined based on intraoperative findings. Kneading of worms toward the cecum was performed in cases with viable bowel and distal obstruction of the terminal ileum. Enterotomy with direct worm extraction was performed when kneading was not feasible or when the obstruction was located at the jejunal level. Bowel resection with primary anastomosis was performed in cases of gangrenous bowel, perforation, or compromised bowel viability.

### Post-operative care and follow-up

Postoperative management consisted of standard surgical care, including close monitoring of vital parameters, wound care, appropriate antibiotic therapy, and gradual resumption of oral feeding following the return of bowel function. Anthelmintic therapy with albendazole (400 mg) was initiated once oral intake was tolerated. A repeat dose was administered at 6 weeks to eradicate parasites that may have been in the larval stage at the time of the initial treatment. All patients remained free of recurrent infestation or disease during follow-up in the outpatient department, with a maximum follow-up period of 3 years.

### Data analysis and outcome measures

Data on patient demographics, clinical presentation, diagnostic findings, treatment modalities, operative procedures, and postoperative complications were systematically collected. The primary outcome measures included the requirement for surgical intervention, type of surgical procedure performed, and postoperative morbidity. Secondary outcomes included length of hospital stay, time to resolution of obstruction with conservative management, and short-term follow-up outcomes.

### Statistical analysis

Data were analyzed using IBM SPSS Statistics for Windows version 26.0 (IBM Corp., Armonk, NY, USA). Continuous variables were summarized as mean ± standard deviation (SD) with range and 95% confidence intervals (CI), while categorical variables were expressed as frequency and percentage. Student’s *t*-test was used to compare continuous variables, and the chi-square test or Fisher’s exact test was applied for categorical variables, as appropriate. Statistical significance was set at *p* < 0.05. Given the primarily descriptive nature of this study and the relatively small sample size, the use of comparative statistical analyses was limited.

## Results

### Demographic and clinical presentation of study population

During the study period, 190 pediatric patients presented to the emergency department with clinical features of intestinal obstruction. Of these, 120 patients (63.2%) were diagnosed with intestinal obstruction secondary to ascariasis, thus representing the most common cause of bowel obstruction in this population.

The mean age of the study group was 5.8 ± 2.3 years. The highest incidence occurred in the 5–8 years group (54.2%; 95% CI 45.3–63.1%), followed by the 2–4 years (37.5%; 95% CI 28.8–46.2%). There was a significant male predominance (66.7%; 95% CI: 58.1–75.3%).The most common presenting symptom was abdominal pain (100%), followed by vomiting (98.3%; 95% CI 95.0–100%), abdominal distension (79.2%; 95% CI 71.9–86.5%), constipation (70.8%; 95% CI 62.4–79.2%), and fever (35.0%; 95% CI 26.2–43.8%). The duration of symptoms prior to presentation ranged from 6 h to 5 days, with most patients presenting within 24–48 h. On physical examination, patients commonly present with varying degrees of abdominal distension, diffuse tenderness, and signs of dehydration in those with prolonged symptoms. Digital rectal examination was unremarkable in most cases, although some patients demonstrated an empty rectum with the absence of stool.

No statistically significant difference was found between sex and the need for surgical intervention (*p* = 0.42, chi-square test). However, younger children (< 8 years) showed a higher frequency of successful conservative management (*p* = 0.03).

The demographic and clinical characteristics of the children with Ascaris-induced intestinal obstruction are summarized in Table 1.

**Table 1:** Demographics and clinical presentation of children with Ascaris-induced intestinal obstruction

Parameter	Number of Patients (*N* = 120)	Percentage (%)	95% CI	Mean ± SD
Age Distribution				
2–4 years	45	37.5	28.8–46.2	
5–8 years	65	54.2	45.3–63.1	
9–12 years	8	6.7	2.2–11.2	
13–16 years	2	1.7	0.0–4.0	
Mean age (years)				5.8 ± 2.3 (95% CI 5.4–6.2)
Gender Distribution				
Male	80	66.7	58.1–75.3	
Female	40	33.3	24.7–41.9	
Clinical Symptoms				
Abdominal pain	120	100	-	
Vomiting	118	98.3	95.0–100.0	
Abdominal distension	95	79.2	71.9–86.5	
Constipation	85	70.8	62.4–79.2	
Fever	42	35	26.2–43.8	

*CI* confidence Interval, *SD* standard Deviation, *N* number of patients

### Diagnostic investigations

Among the 120 children, leucocytosis (> 11,000/µL) was observed in 45 patients (37.5%; 95% CI: 28.8–46.2%), anaemia (Hb < 10 g/dL) in 38 (31.7%; 95% CI: 23.3–40.1%), and eosinophilia in 11 (9.2%; 95% CI: 3.9–14.5%). Electrolyte imbalance was documented in 52 cases (43.3%; 95% CI: 34.3–52.3%). Stool examination was performed in 78 patients and revealed *Ascaris* ova in 65 (83.3%; 95% CI: 74.7–91.9%).

On imaging, abdominal X-ray demonstrated visible worm shadows in 28 patients (23.3%; 95% CI: 15.5–31.1%). Ultrasonography detected intestinal ascariasis in 82 of 85 confirmed cases, yielding a sensitivity of 96.5% (95% CI: 90.1–99.3). The positive predictive value was 100% (95% CI: 95.6–100). Specificity and negative predictive value could not be calculated due to the absence of true-negative cases in the study population. Abdominal CT was performed in 12 selected cases and was diagnostic in all (100%).

The laboratory and imaging investigations in children with Ascaris-induced intestinal obstruction are summarized in Table 2.

**Table 2:** Laboratory and imaging investigations in children with Ascaris-induced intestinal obstruction

Investigation	Number of Cases	Positive/Abnormal Results	Percentage (%)	95% CI
Laboratory Tests				
Complete Blood Count	120	-	-	-
- Leukocytosis (> 11,000/µL)	120	45	37.5	28.8–46.2
-Eosinophilia	120	11	9.2	3.9–14.5
- Anemia (Hb < 10 g/dL)	120	38	31.7	23.3–40.1
Electrolyte imbalance	120	52	43.3	34.3–52.3
Stool Examination	78	65	83.3	74.7–91.9
Imaging Studies				
Plain Abdominal X-ray	120	-	-	-
- Visible worm shadows	120	28	23.3	15.5–31.1
Abdominal Ultrasound	85	82	96.5	93.1–99.8
CT Scan Abdomen	12	12	100	-

*CI* confidence interval

### Management strategies and outcomes

Of the 120 children with ascaris-induced intestinal obstruction, 80 (66.7%; 95% CI: 58.1–75.3%) were initially managed conservatively, and 40 (33.3%; 95% CI: 24.7–41.9%) underwent immediate surgical intervention. Among the conservatively managed patients, 65 (81.3%; 95% CI: 72.7–89.8%) responded successfully without operative treatment, whereas 15 (18.7%; 95% CI: 10.2–27.3%) failed conservative therapy and required surgery. Thus, a total of 55 children (45.8%; 95% CI: 36.9–54.7%) ultimately required operative management.

The success rate of conservative management was significantly higher in patients aged < 8 years (*p* = 0.03), while older children were more likely to require surgery. No sex-based differences were observed in treatment outcomes (*p* = 0.41).

The management approach and treatment outcomes in children with Ascaris-induced intestinal obstruction are summarized in Table 3.

**Table 3:** Management approach and treatment outcomes in children with Ascaris-induced intestinal obstruction

Management Approach	Number of Cases (*N* = 120)	Percentage (%)	95% CI
Initial Conservative Management	80	66.7	56.1–75.3
- Successful without surgery	65	81.3	72.7–89.8
- Failed, required surgery	15	18.7	10.2–27.3
Immediate Surgical Intervention	40	33.3	24.7–41.9
Total Surgical Cases	55	45.8	36.9–54.7

*CI* confidence Interval, *N* number of patients

### Surgical management

Among the 55 children who underwent surgery, the most common procedure was kneading of worms into the colon in 20 patients (36.4%; 95% CI: 23.5–49.3%), followed by enterotomy with worm extraction in 18 patients (32.7%; 95% CI: 20.3–45.1%) and bowel resection with end-to-end anastomosis in 17 patients (30.9%; 95% CI: 18.7–43.1%).

Children who required bowel resection generally presented late with features of gangrenous or perforated bowel disease. The mean hospital stay progressively increased with the complexity of the surgical procedure: 5.7 ± 1.6 days for kneading, 7.3 ± 1.8 days for enterotomy, and 10.6 ± 2.4 days for bowel resection and anastomosis (*p* < 0.001, ANOVA test).

The surgical procedures performed for ascariasis-induced intestinal obstruction are summarized in Table 4.

**Table 4:** Types of surgical procedures performed and corresponding mean hospital stay in children with Ascaris-induced intestinal obstruction

Surgical Procedure	Number of Cases (*n* = 55)	Percentage (%)	95% CI	Mean Hospital Stay (Days ± SD)
Kneading of worms into the colon	20	36.4	23.5–49.3	5.7 ± 1.6
Enterotomy with worm extraction	18	32.7	20.3–45.1	7.3 ± 1.8
Bowel resection with anastomosis	17	30.9	18.7–43.1	10.6 ± 2.4

*CI* confidence Interval, *SD* standard Deviation, *n* number of patients

**Fig. 1:**
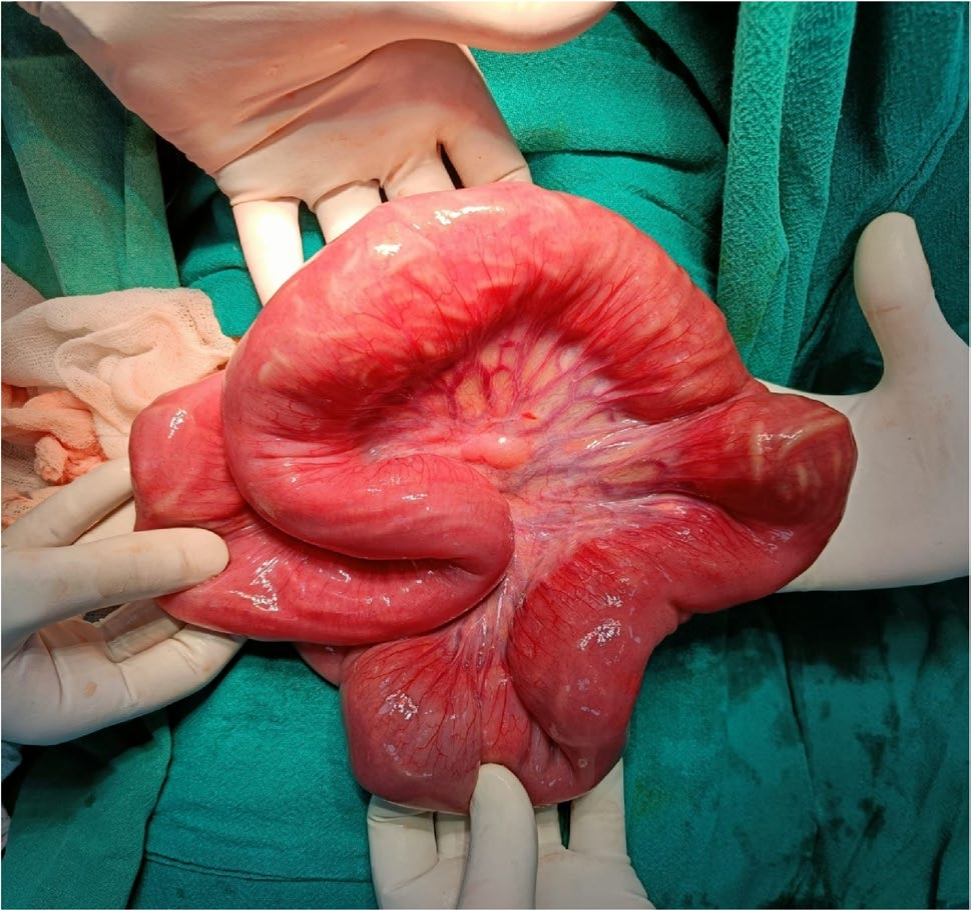
Intraoperative image of a patient with a huge worm load

Figure 1 shows an intraoperative picture of a patient with a huge worm load. The patient was successfully managed with enterotomy and worm extraction. The postoperative course was uneventful, and the patient was discharged uneventfully.

**Fig. 2:**
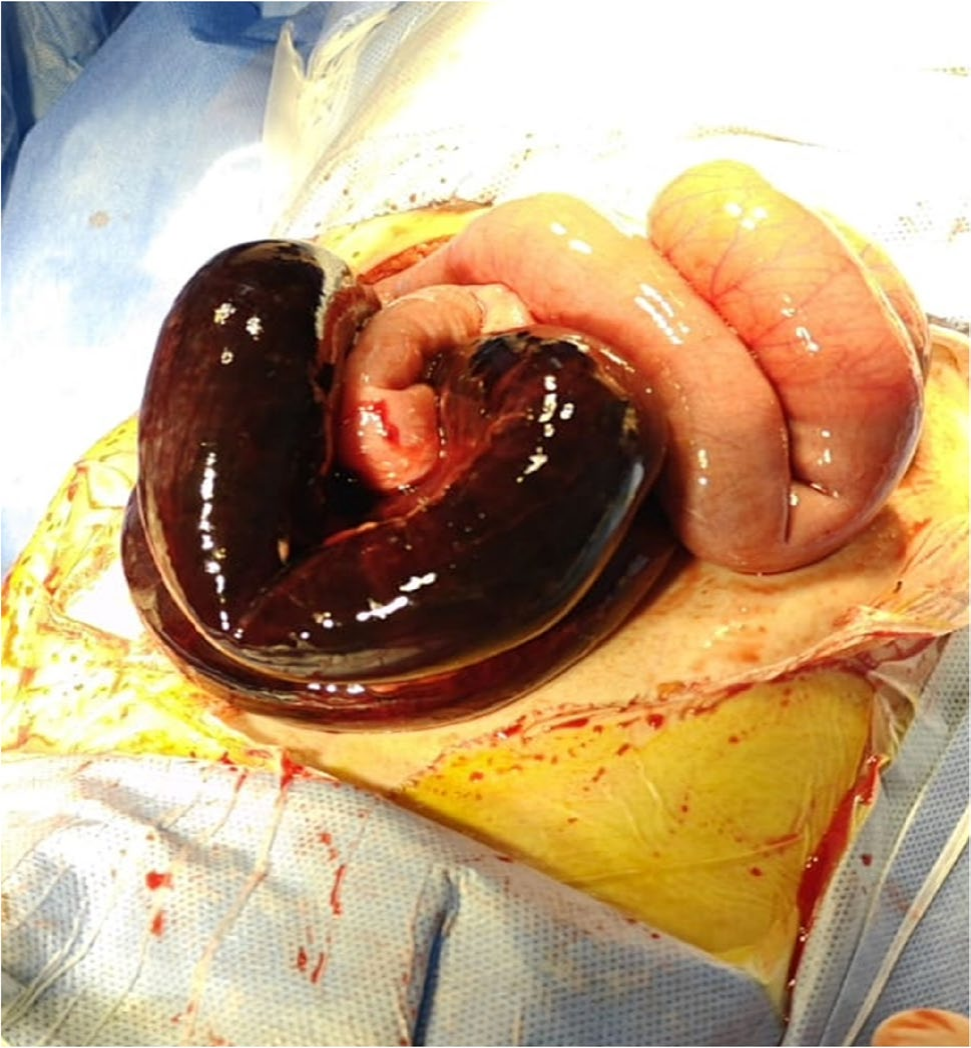
Intra-operative picture of a patient with worm induced segmental volvulus/gangrene

Figure 2 shows an intraoperative picture of a patient with worm induced segmental volvulus of small bowel. The patient was successfully managed with resection of the gangrenous ileal segment and end-end ileo-ileal anastomosis. The postoperative course was uneventful, and the patient was discharged uneventfully.

### Surgical complications and morbidity

Postoperative complications occurred in 8 of 55 surgical patients (14.5%; 95% CI: 5.4–23.7%), while 47 (85.5%; 95% CI: 76.3–94.6%) had uneventful recovery. The most common complication was surgical site infection (SSI) in four patients (7.3%; 95% CI: 0.4–14.2%), all managed conservatively with dressings and antibiotics. Anastomotic leak occurred in two patients (3.6%; 95% CI: 0.0–8.5%); one was managed conservatively and the other required re-exploration. During re-exploration, worms were found protruding through the anastomotic site. Burst abdomen was seen in two patients (3.6%; 95% CI: 0.0–8.5%), both of whom were managed with secondary suturing. No mortality was recorded in the present series, demonstrating the effectiveness of timely intervention and appropriate management.

The mean hospital stay was significantly longer in patients with complications (14.8 ± 3.5 days) than in those without complications (7.2 ± 1.6 days, *p* < 0.001, Student’s *t*-test).

The postoperative complications and their management in surgically treated patients are summarized in Table 5.

**Table 5:** Postoperative complications and their management following surgery for Ascaris-induced intestinal obstruction

Complication	Number of Cases (*n* = 55)	Percentage (%)	95% CI	Management	Clavien–Dindo Classification
Surgical site infection	4	7.3	0.4–14.2	Conservative	Grade-Ⅱ
Anastomotic leak	2	3.6	0.0–8.5.0.5	1 Conservative, 1 Re-exploration	Grade-Ⅱ (Conservative) Grade-Ⅲb (Re-exploration)
Burst abdomen	2	3.6	0.0–8.5.0.5	Secondary suture	Grade-Ⅲb
Total complications	8	14.5	5.4–23.7	-	-
No complications	47	85.5%	76.3–94.6	-	-
Mortality	0	0	-	-	-

*CI* confidence Interval, *n* number of patients

### Treatment outcomes and recovery

**Fig. 3:**
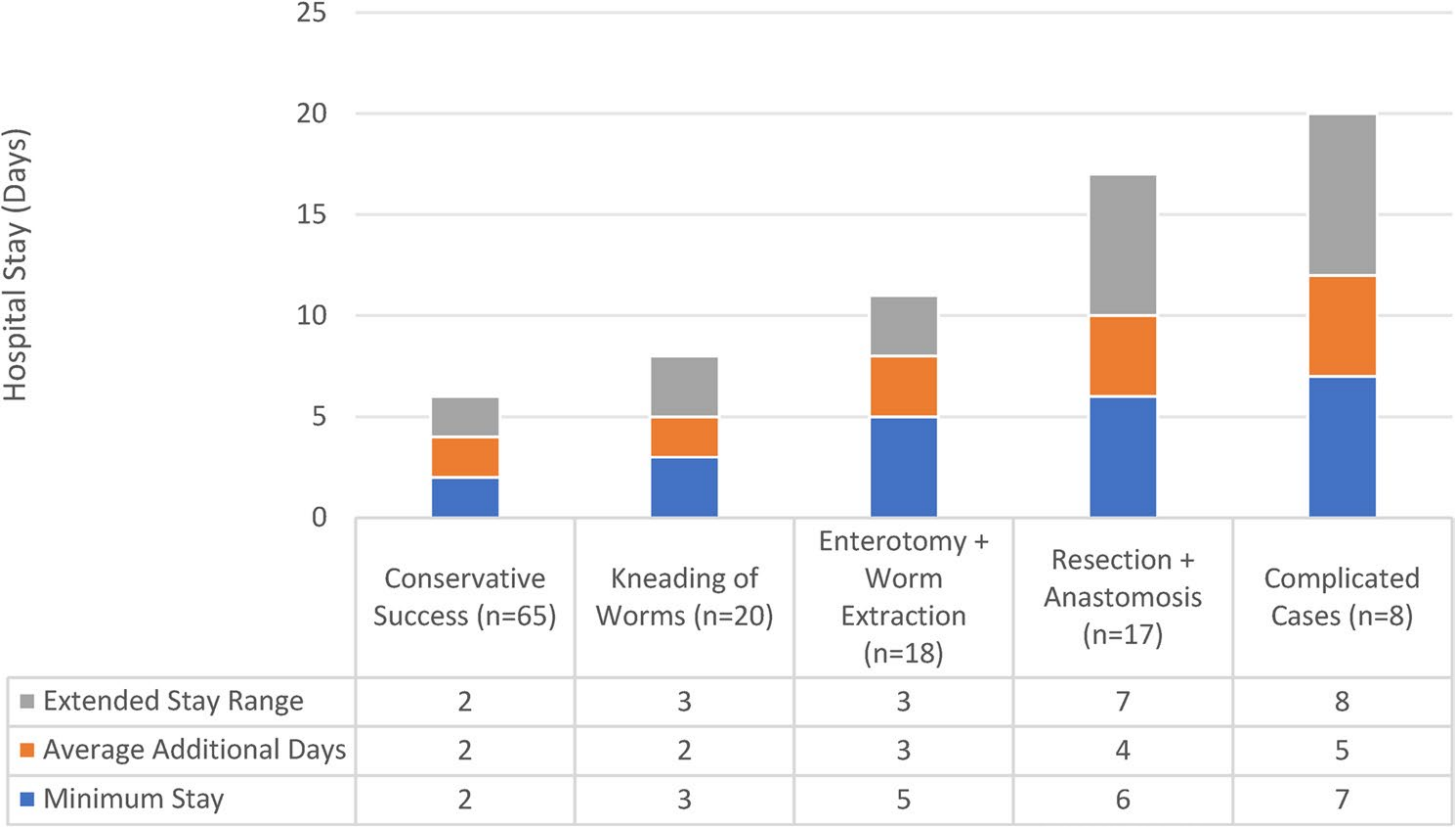
Hospital Stay Duration Distribution by Management Type

Patients managed conservatively (*n* = 65) had the shortest hospital stay (mean 4.2 ± 1.5 days; range 2–6 days). Those who underwent worm kneading (*n* = 20) had a mean hospital stay of 5.7 ± 1.6 days (range 3–8 days), while enterotomy with worm extraction (*n* = 18) was associated with a mean duration of 7.3 ± 1.8 days (range 5–11 days). Patients requiring bowel resection and end-to-end anastomosis (*n* = 17) had longer hospitalization (10.6 ± 2.4 days; range 6–17 days), and complicated cases (*n* = 8) had the longest stays (14.8 ± 3.5 days; range 7–20 days), as shown in Fig. 3.

## Discussion

Our study demonstrates that intestinal ascariasis remains a major cause of pediatric surgical emergencies in endemic regions, accounting for 63.2% of cases of intestinal obstruction. This is consistent with reports from other developing countries, where ascariasis is the leading cause of bowel obstruction in children, in contrast to developed nations, where postoperative adhesions predominate [22, 23].

A male predominance of 2:1 was observed, consistent with previous studies, although the underlying reason remains unclear and may be related to behavioral factors influencing exposure to contaminated environments [24]. The majority of cases (91.7%) occurred in children aged 2–8 years, supporting the understanding that younger children are at higher risk due to their smaller intestinal diameter and behaviors that increase contact with infective ova [25, 26].

Abdominal pain was a universal presenting symptom (100%), with vomiting reported in 98.3% of cases, mirroring the findings of Villamizar et al. [4]. Fever was present in 35% of patients, potentially reflecting heightened inflammatory responses to the worm burden or more advanced cases with complications.

### Diagnostic accuracy and imaging

The diagnostic yield of ultrasonography in our series was 96.5% among scanned patients, exceeding the rates reported in earlier studies, likely reflecting improved operator expertise and equipment quality. Characteristic sonographic signs of ascariasis, including the “bull’s eye” and “railway track” appearances, are reliable for diagnosis [17, 18]. In contrast, visible worm shadows on plain radiographs were noted in only 23.3% of cases, consistent with previous reports, highlighting the limitations of conventional radiography in detecting intestinal ascariasis [13].

### Management outcomes and surgical intervention

The success rate of conservative management in our series was 81.3%, comparable to previous reports by Wasadikar and Kulkarni (78%) and Hefny et al. (75–80%) [1, 14], supporting the use of initial conservative therapy in patients without peritoneal signs or complete obstruction. The overall rate of surgical intervention was 45.8%, which was higher than that in some reports, likely reflecting the tertiary care setting and referral patterns. Among the surgical procedures, worm kneading was the most common (36.4%), followed by enterotomy with worm extraction (32.7%) and bowel resection with primary anastomosis (30.9%), illustrating the spectrum of operative complexity in these patients [15, 19].

### Surgical complications and novel findings

The overall postoperative complication rate was 14.5%, which is within the acceptable limits for emergency pediatric abdominal surgery. Anastomotic leaks occurred in 3.6% of cases, aligning with published series. Notably, live worms emerging from an anastomotic site were observed, underscoring their parasitic behavior and supporting the hypothesis that worms actively seek areas of weakness [21, 27]. No mortality was recorded, likely reflecting timely surgical intervention, optimized perioperative care, and improved understanding of disease pathophysiology, in contrast to earlier regional reports citing mortality rates of 2–5% [16].

### Contemporary relevance and prevention

Despite advances in socioeconomic conditions and public health measures, the high prevalence of ascariasis-related intestinal obstruction in our series highlights the continued burden of this disease in endemic regions. Anti-helminthic therapy was highly effective, achieving a 96.4% clearance rate and reinforcing the need for comprehensive management that addresses both acute obstruction and underlying parasitic infections [28, 29]. The absence of recurrent obstruction in our follow-up cohort emphasizes the importance of patient education and hygiene counselling. Long-term control of this preventable condition relies on mass deworming programs and improved sanitation [30].

### Limitations and future directions

This study had several limitations. The single-center, tertiary referral cohort may limit the generalizability of the findings and introduce referral bias, as patients are often referred with advanced disease, increasing the likelihood of failed conservative management and the need for surgery. The follow-up period was relatively short, potentially underestimating the long-term complications or recurrence. Additionally, the high diagnostic accuracy of ultrasonography may not be generalizable because of operator dependency. Future multicenter studies with extended follow-ups are warranted to provide more robust data on outcomes and preventive strategies.

### Clinical implications

This study has several important clinical implications. The high prevalence of ascariasis-induced intestinal obstruction in endemic regions underscores the need for a high index of suspicion in pediatric patients presenting with such obstruction. The demonstrated success of conservative management in selected cases supports initial non-operative approaches, whereas favorable surgical outcomes highlight that timely operative intervention achieves excellent results with minimal morbidity. The findings also emphasize the value of a multidisciplinary approach, integrating emergency care, pediatric surgery, and public health measures, with coordinated parasitic control programs offering the most effective strategy for reducing the burden of this preventable condition.

## Conclusion

In this cohort from an endemic region, intestinal ascariasis was the leading cause of pediatric intestinal obstruction, underscoring its continued burden. This condition remains a significant surgical challenge, necessitating early recognition and timely, appropriate management to optimize outcomes. Despite favorable surgical results, the persistently high prevalence highlights the urgent need for effective preventive strategies and sustained public health interventions to reduce this avoidable cause of pediatric morbidity in the future.

## Data Availability

The datasets used and/or analyzed during the current study are included in the manuscript and are available from the corresponding author on reasonable request.

## References

[1] Hefny AF, Saadeldin YA, Abu-Zidan FM. Management algorithm for intestinal obstruction due to ascariasis: a case report and review of the literature. Ulus Travma Acil Cerrahi Derg. 2009;15(3):301–5. 19562557

[2] Bethony J, Brooker S, Albonico M, Geiger SM, Loukas A, Diemert D, et al. Soil-transmitted helminth infections: ascariasis, trichuriasis, and hookworm. Lancet. 2006;367:1521–32. 16679166 10.1016/S0140-6736(06)68653-4

[3] de Silva NR, Guyatt HL, Bundy DA. Morbidity and mortality due to Ascaris-induced intestinal obstruction. Trans R Soc Trop Med Hyg. 1997;91:31–6. 9093623 10.1016/s0035-9203(97)90384-9

[4] Villamizar E, Méndez M, Bonilla E, Varon H, de Onatra S. *Ascaris lumbricoides* infestation as a cause of intestinal obstruction in children: experience with 87 cases. J Pediatr Surg. 1996;31:201–4. 8632280 10.1016/s0022-3468(96)90348-6

[5] Bar Moar JA, de Carvalho JL, Chappell J. Use of gastrografin in sub-acute intestinal obstruction due to *Ascaris lumbricoides*. J Pediatr Surg. 1984;19:174–6. 6726573 10.1016/s0022-3468(84)80442-x

[6] Wani SA, Ahmad F, Zargar SA, Dar ZA, Dar PA, Tak H, Fomda BA. Soil-transmitted helminths in relation to hemoglobin status among school children of the Kashmir Valley. J Parasitol. 2008;94(3):591–3. 18605794 10.1645/GE-1400.1

[7] Louw JH. Abdominal complications of *Ascaris lumbricoides* infestation in children. Br J Surg. 1966;53:510–21. 5936979 10.1002/bjs.1800530606

[8] Dayalan N, Ramakrishnan MS. The pattern of intestinal obstruction with special preference to ascariasis. Indian Pediatr. 1976;13(1):47–9. 1278946

[9] Archibong AE, Ndoma-Egba R, Asindi AA. Intestinal obstruction in southeastern Nigerian children. East Afr Med J. 1994;71:286–9. 7925057

[10] Khuroo MS. Ascariasis. Gastroenterol Clin North Am. 1996;25:553–77. 8863040 10.1016/s0889-8553(05)70263-6

[11] Warren KS, Mahmoud AA. Algorithms in the diagnosis and management of exotic diseases. J Infect Dis. 1977;135:868–72. 858948 10.1093/infdis/135.5.868

[12] Wiersma R, Hadley GP. Small bowel volvulus complicating intestinal ascariasis in children. Br J Surg. 1988;75:86–7. 3337961 10.1002/bjs.1800750130

[13] Coflkun A, Ozcan N, Durak AC, Tolu I, Gülec M, Turan C. Intestinal ascariasis as a cause of bowel obstruction in two patients: sonographic diagnosis. J Clin Ultrasound. 1996;24:326–8. 8792275 10.1002/(SICI)1097-0096(199607/08)24:6<326::AID-JCU9>3.0.CO;2-K

[14] Wasadikar PP, Kulkarni AB. Intestinal obstruction due to ascariasis. Br J Surg. 1997;84:410–2. 9117326

[15] Mukhopadhyay B, Saha S, Maiti S, Mitra D, Banerjee TJ, Jha M, et al. Clinical appraisal of *Ascaris lumbricoides*, with special reference to surgical complications. Pediatr Surg Int. 2001;17:403–5. 11527176 10.1007/s003830000517

[16] Thein-Hlaing. A profile of ascariasis morbidity in Rangoon children’s Hospital, Burma. J Trop Med Hyg. 1987;90:165–9. 2958641

[17] Chawla A, Patwardhan V, Maheshwari M, Wasnik A. Primary ascaridial perforation of the small intestine: sonographic diagnosis. J Clin Ultrasound. 2003;31:211–3. 12692830 10.1002/jcu.10152

[18] Mahmood T, Mansoor N, Quraishy S, Ilyas M, Hussain S. Ultrasonographic appearance of *Ascaris lumbricoides* in the small bowel. J Ultrasound Med. 2001;20:269–74. 11270532 10.7863/jum.2001.20.3.269

[19] Ahmed S, Naqvi SA, Phulpoto MA. Surgical management of intestinal obstruction due to ascariasis in children. J Pak Med Assoc. 1999;49:323–5.

[20] Saha ML, Chakrabarti P, Ghosh P, Chattopadhyay A, Choudhury CR. Ileocolic intussusception due to *Ascaris lumbricoides*. Indian J Pediatr. 1993;60:561–3.

[21] Reeder MM, Palmer PES. The radiology of tropical diseases with epidemiological, pathological and clinical correlation. 4th ed. Baltimore: Williams & Wilkins; 1998.

[22] Hotez PJ, Bundy DAP, Beegle K, Brooker S, Drake L, de Silva N, et al. Helminth infections: soil-transmitted helminth infections and schistosomiasis. In: Jamison DT, Breman JG, Measham AR, et al. editors. Disease control priorities in developing countries. 2nd ed. Washington (DC): The International Bank for Reconstruction and Development / The World Bank; New York Oxford Press; 2006. Chapter 24. Bookshelf ID: NBK11748; PMID: 21250326. 21250326

[23] Crompton DWT. *Ascaris* and ascariasis. Adv Parasitol. 2001;48:285–375. 11013758 10.1016/s0065-308x(01)48008-0

[24] Brooker S, Bethony J, Hotez PJ. Human hookworm infection in the 21st century. Adv Parasitol. 2004;58:197–288. 15603764 10.1016/S0065-308X(04)58004-1 PMC2268732

[25] O’Lorcain P, Holland CV. The public health importance of *Ascaris lumbricoides*. Parasitology. 2000;121:S51-71. 11386692 10.1017/s0031182000006442

[26] Chan MS. The global burden of intestinal nematode infections—fifty years on. Parasitol Today. 1997;13:438–43. 15275146 10.1016/s0169-4758(97)01144-7

[27] Ochoa B. Surgical complications of ascariasis. World J Surg. 1991;15:222–7. 2031358 10.1007/BF01659056

[28] Keiser J, Utzinger J. Efficacy of current drugs against soil-transmitted helminth infections: systematic review and meta-analysis. JAMA. 2008;299:1937–48. 18430913 10.1001/jama.299.16.1937

[29] Vercruysse J, Behnke JM, Albonico M, Ame SM, Angebault C, Bethony JM, et al. Assessment of the anthelmintic efficacy of albendazole in school children in seven countries where soil-transmitted helminths are endemic. PLoS Negl Trop Dis. 2011;5(3):e948. 21468309 10.1371/journal.pntd.0000948 PMC3066140

[30] Hotez PJ, Molyneux DH, Fenwick A, Kumaresan J, Sachs SE, Sachs JD, et al. Control of neglected tropical diseases. N Engl J Med. 2007;357:1018–27. 17804846 10.1056/NEJMra064142

